# Uptake of premarital HIV testing and associated factors among women who had autonomous versus non autonomous marriage in Ethiopia: A nationwide study

**DOI:** 10.1371/journal.pone.0271879

**Published:** 2022-08-18

**Authors:** Mohammed Ahmed, Seada Seid, Ali Yimer, Abdu Seid, Ousman Ahmed

**Affiliations:** 1 Department of Public Health, College of Health Science, Woldia University, Woldia, Ethiopia; 2 Department of Midwifery, College of Health Science, Woldia University, Woldia, Ethiopia; 3 Department of Nursing, College of Health Science, Woldia University, Woldia, Ethiopia; Oregon State University, UNITED STATES

## Abstract

**Background:**

Premarital HIV testing offers an opportunity where prospective couples can know their HIV status before marriage to prevent both heterosexual and vertical transmission of HIV. Therefore, this study aimed to determine whether there is any significant difference in the prevalence of premarital HIV testing among women who had autonomous versus non-autonomous marriage, and to investigate the factors associated with premarital HIV testing among women who had autonomous versus non-autonomous marriage in Ethiopia.

**Methods:**

Data were extracted from 2016 Ethiopia Demographic and Health Survey dataset and analyzed by using SPSS version 20. Frequencies and weighted percentage of the variables, and second-order Rao-Scott statistic were computed. Multivariable logistic regression analysis was used to identify factors between the two groups. An adjusted odds ratio with 95% confidence interval was considered to state statistically significant associations.

**Result:**

From 9602 included sample, 4,043 (42.1%) of the women had autonomous marriage, and 5,559(57.9%) of the women had non-autonomous marriage. The prevalence of premarital HIV testing in Ethiopia among women who had autonomous marriage was 30.5% (95% CI: 27.7–33.4%) compared to 20.6% (95% CI: 18.5–22.8) among women who had a non-autonomous marriage. No differences in associated factors were found between women who had autonomous versus non autonomous marriage to uptake HIV testing. In both groups, residence in rural area, education attainment (primary, secondary, higher), media access, being rich and richest, knowing the places for HIV testing, chewing chat, and drinking alcohol were significantly predicts premarital HIV testing.

**Conclusion:**

The study indicated that 10% more women in autonomous marriage tested for HIV relative to non-autonomous women whilst being an urban resident, educated, having access to media, household wealth and knowledge of testing facilities significantly predict HIV testing among women in Ethiopia. The paper recommends the Ethiopian government shall expand access to education among women while improving their access to media to enhance their socioeconomic wellbeing and health. Furthermore, it is better to inspire women to undergo autonomous marriage by fostering education in the community to enhance premarital HIV testing.

## Introduction

Women are particularly vulnerable to HIV infection because of increased biological susceptibility to HIV transmission through heterosexual contact [1, 2], and faced a host of structural barriers and contextual gender inequalities [3, 4]. Compared to men, women living in Sub-Saharan Africa (SSA) are more affected by HIV, accounting for 59% of all infections in this region [5]. In Ethiopia, HIV prevalence varies notably by marital status and is 0.8% higher among women who report ever having been married compared with those who have never married (0.3%) [6].

For this reason, HIV testing for marriage applicants is recommended in Africa since it has been acknowledged as a renowned and cost-effective measure [7, 8], as well as a convenient means of HIV infection surveillance [9]. Premarital HIV testing offers an opportunity where prospective couples can know their HIV status before marriage [10] to prevent both heterosexual and vertical transmission of HIV [11–13]. Pieces of evidence showed that about 50–85% of new infections among married/cohabiting partner was due to HIV sero-discordant couples [14, 15], which results in increased risk for HIV negative partners [16].

Recently, many countries including the government of Ethiopia have initiated premarital HIV testing [10, 17], since it is one of the key elements in the prevention and control of HIV/AIDS in the country [10, 18]. According to the 2016 Ethiopia Demographic Health Survey (EDHS) report, 24.5% of married women aged 15–49 ever tested before getting married or living with a partner [19]. A previous study done in Ethiopia among married women showed that being urban residents, attended education, access to media, improved wealth index, known the place of HIV testing, having the discriminatory attitude to a patient living PLHIV, being khat chewer, and alcohol drinker was significantly associated with premarital HIV testing [20]. The above mentioned study merely focused on the factors affecting premarital HIV testing among married women without considering the marital status whether the marriage was autonomous (women who accept marriage proposals on their own volition and without coercion) versus non-autonomous marriage (women whose marriages were decided by partner, families or relatives, and others). Different literature showed that non-autonomous marriage has considerable detrimental health and social consequences such as it exposes them to a lifetime of domestic violence and abuse as they lack standing and power within their households [21], gynecological problems [22], sexually transmitted infections, and psychological and mental disorders [23].

Given this identified gap in the literature, the purpose of this analysis were: 1) To determine whether there is any significant difference in the prevalence of premarital HIV testing among women who had autonomous versus non-autonomous marriage, 2) To investigate the factors associated with premarital HIV testing among women who had autonomous versus non-autonomous marriage in Ethiopia to tailor specific intervention for the potential delineated factors to hinder HIV transmission.

## Methods and materials

### Data

The current study uses secondary data from the 2016 Ethiopia Demographic Health Survey (EDHS). The 2016 EDHS sample is stratified and was selected in two stages. Each region was stratified into urban and rural areas, which yielded 21 sampling strata. In the first stage, 645 Enumeration areas (EAs) were selected with probability proportional to the EA size and with independent selection in each sampling stratum with the sample allocation. In the second stage of selection, a fixed number of 28 households per cluster were selected with an equal probability systematic selection from the newly created household listing. Thus, a 2016 EDHS cluster is either an EA or a segment of an EA. Based on a fixed sample take of 28 households per cluster, the survey selected 645 EAs, 202 in urban areas and 443 in rural areas. The survey was conducted in 16,650 residential households, A detailed description of the study design and methodology of 2016 EDHS is found in the report [19]. This study was based on the woman questionnaire, which was administered to 15, 683 Ethiopian women aged 15–49 in the selected households.

### Selection criteria

**Fig 1:**
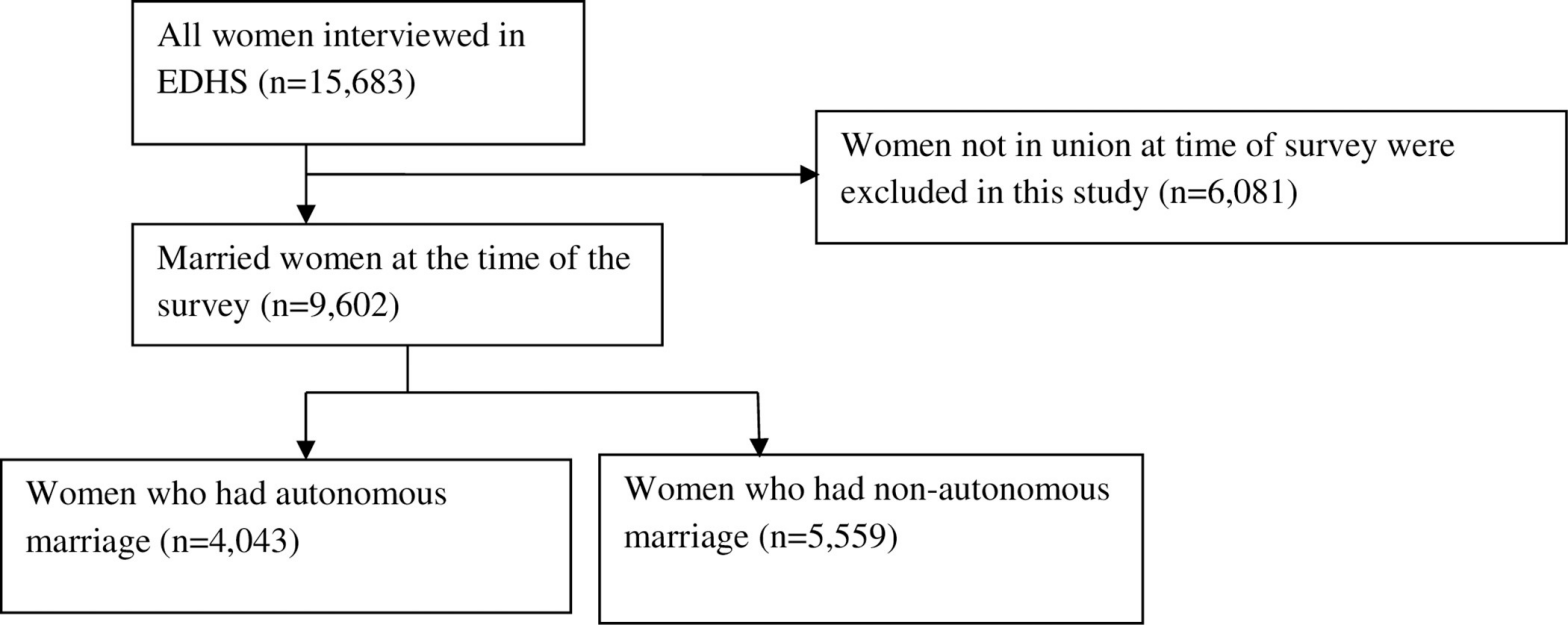
Flow chart showing the weighted sample used in the study was derived.

The sample utilized in this study excluded women who were not in a marital union (*n* = 6,081). The analytic sample for the current study consisted of 9,602 married women. From the included sample, 4,043 of the women had autonomous marriage, and 5,559 of the women had non-autonomous marriage (**Fig 1**).

### Study variables

The dependent variable of the study was a self-reported history of premarital HIV testing among women who had autonomous versus non autonomous marriage. Respondents were asked as “did you undertake an HIV test before you got married?” The responses options were “Yes” or “No”.

In this study, marriage autonomy was assessed by asking the question in DHS as ‘the first time you got married who decide on your marriage?. The questions had the following responses; by myself, parents, other family/ relatives, and others. The responses were coded as women had autonomous marriage if the women decided their marriage by themselves, and women had non-marriage if the women decided their marriage by parents, other family/ relatives, and others.

The independent variables were selected based on a literature review which deemed to be the factors associated with premarital HIV testing and includes age, education status, type of residence, occupation, wealth index (poorest, poorer, middle, richer and richest), media access, knowing the places for HIV testing, comprehensive knowledge about HIV, khat chewing, and alcohol drinking (yes/ no).

*Comprehensive knowledge about HIV/AIDS* was defined based on a widely used measure where each woman was asked whether or not she agreed or disagreed with the following five items: (1) Consistent use of condoms during sexual intercourse can reduce the chance of getting HIV;(2) having just one uninfected faithful partner can reduce the chance of getting HIV; (3) Healthy-looking person can have HIV; (4) HIV can be transmitted by mosquito bites; and (5) a person can become infected by sharing food with a person who has HIV. An additive summary score was created and which was then dichotomized to create a binary variable with 0 indicating at least one incorrect response and 1 to indicate correct response to five items.

*Access to media* was defined based on response to how often respondents read a newspaper, listened to the radio, or watched television. Respondents were asked: “How often do you, read a newspaper, listen to the radio, or watch TV in a week?”. Those who responded at least once a week to any of these sources were considered to have access to media/media exposure.

### Statistical analysis

The data were analyzed by using Statistical Package for Social Science (SPSS) version 20. All statistical procedures incorporated complex sampling design analysis applied in the 2016 EDHS. Frequencies and weighted percentage of study variables were calculated. Rao–Scott chi-square test was used to examine the relationship between premarital HIV testing and each of the independent variables separately for women who had autonomous versus non-autonomous marriage. Multivariable binary logistic regression analysis was performed to control confounders and to identify independent factors about premarital HIV testing among each group. All independent variables were entered in the multivariable logistic regression model irrespective of the p-values in the bivariate analysis. Adjusted odds ratio (AOR) with a 95% confidence interval and 2-sided p-value was used to state statistically significant associations. As recommended for complex survey design, sampling weights were applied for this study by dividing the individual women sample weight by 1000,000.

### Ethics approval and consent to participate

Ethical clearance for the study was not required since it was a secondary data analysis from EDHS 2016 database. The researchers had received the survey data from USAID–DHS program and then the researchers of this study have maintained the confidentiality of the data.

## Result

### Socio-demographic characteristics of study participants by type of marriage

From the included sample, 4,043 (42.1%) of the women had autonomous marriage, and 5,559(57.9%) of the women had non-autonomous marriage. Among women who had autonomous marriage, the proportion of women who were reside in rural area was 72.8%. Besides, 28% of the study participants were found in the age range of 25–29 years. The participants who didn’t attend education shared 44.2%. Moreover, 96.7% of the respondent didn’t have comprehensive knowledge about HIV **(Table 1).**

**Table 1:** Characteristics of the study participants who had autonomous marriage (n = 4043).

Variables	Category	Overall	Premarital HIV testing among women who had autonomous marriage		p-value*
			No	Yes	
		n(wt.%)	n (wt. %)	n (wt. %)	
Residence	Urban	1647(27.2)	714(15.4)	933(54.0)	P<0.001
	Rural	2396(72.8)	1979(84.6)	417(46.0)	
Age	15–19	290(7.2)	193(7.4)	97(6.8)	p<0.001
	20–24	877(22.1)	503(18.6)	374(29.9)	
	25–29	1059(27.9)	653(26.0)	406(32.2)	
	30–34	746(18.5)	490(18.5)	256(18.5)	
	35–39	547(12.2)	417(14.2)	130(7.6)	
	40–44	337(7.6)	278(9.4)	59(3.5)	
	45–49	187(4.5)	159(5.9)	28(1.4)	
Educational status	No education	1731(44.2)	1554(56.9)	177(15.3)	p<0.001
	Primary	1273(35.2)	769(32.6)	504(41.0)	
	Secondary	591(11.3)	245(6.4)	346(22.3)	
	Higher	448(9.3)	125(4.1)	323(21.4)	
Occupation	Unemployed	2314(52.9)	1701(56.8)	613(44.0)	p<0.001
	Agricultural	364(12.8)	286(14.3)	78(9.2)	
	Non-agricultural	1365(34.4)	706(28.9)	659(46.8)	
Access to media	No	2042(54.7)	1786(67.1)	256(26.4)	p<0.001
	Yes	2001(45.3)	907(32.9)	1094(73.6)	
Wealth index	Poorest	937(16.0)	856(20.6)	81(5.5)	p<0.001
	Poorer	468(16.9)	397(20.7)	71(8.3)	
	Middle	453(16.7)	368(19.6)	85(10.0)	
	Richer	459(17.4)	338(18.1)	121(15.9)	
	Richest	1726(33.0)	734(21.0)	992(60.4)	
Comprehensive knowledge about HIV	No	3972(96.7)	2634(96.0)	1338(98.3)	0.028
	Yes	71(3.3)	59(4.0)	12(1.7)	
Know the places to HIV testing	No	566(20.8)	535(29.1)	31(3.7)	p<0.001
	Yes	3049(79.2)	1747(70.9)	1302(96.3)	
Chewing khat	No	3515(87.1)	2353(86.2)	1162(89.1)	
	Yes	528(12.9)	340(13.8)	188(10.9)	0.183
Alcohol drinking	No	3040(75.1)	2230(81.1)	810(61.4)	p<0.001
	Yes	1003(24.9)	463(18.9)	540(38.6)	

Similarly, among women who had non-autonomous marriages, the proportion of women who were reside in rural area was 90.4%. Besides, 21.1% of the study subjects were found in the age range of 25–29 years. The participants who didn’t attend education shared 71.4%. Moreover, 96.8% of the respondents didn’t have comprehensive knowledge about HIV **(Table 2).**

**Table 2:** Characteristics of the study participants who didn’t have autonomous marriage (n = 5559).

Variables	Category	Overall	Premarital HIV testing among women who had not autonomous marriage		p-value
			No	Yes	
		n(wt.%)	n (wt. %)	n (wt. %)	
Residence	Urban	722(9.6)	395(6.0)	327(23.3)	<0.001
	Rural	4837(90.4)	4054(94.0)	783(76.7)	
Age	15–19	351(4.8)	240(4.0)	111(8.1)	<0.001
	20–24	848(13.5)	588(11.8)	260(20.2)	
	25–29	1130(21.1)	817(18.8)	313(30.3)	
	30–34	1068(21.0)	865(21.4)	203(19.4)	
	35–39	989(17.8)	872(19.5)	117(11.6)	
	40–44	669(12.0)	611(13.8)	58(5.4)	
	45–49	504(9.6)	456(10.8)	48(5.0)	
Educational status	No education	3894(71.4)	3394(76.8)	500(50.6)	<0.001
	Primary	1348(24.3)	936(21.4)	412(35.3)	
	Secondary	248(3.4)	93(1.6)	155(10.5)	
	Higher	69(0.9)	26(0.2)	43(3.6)	
Occupation	Unemployed	2959(51.5)	2416(52.2)	543(48.8)	<0.001
	Agricultural	1526(29.4)	1294(30.7)	232(24.5)	
	Non-agricultural	1074(19.1)	739(17.1)	335(26.7)	
Access to media	No	3757(66.2)	3239(70.8)	518(48.6)	<0.001
	Yes	1802(33.8)	1210(29.2)	592(51.4)	
Wealth index	Poorest	1943(21.0)	1742(23.2)	201(12.4)	<0.001
	Poorer	1006(22.3)	851(23.9)	155(16.4)	
	Middle	889(22.2)	715(22.7)	174(20.4)	
	Richer	830(20.8)	645(20.3)	185(22.7)	
	Richest	891(13.7)	496(9.9)	395(28.1)	
Comprehensive knowledge about HIV	No	5424(96.8)	4340(96.8)	1084(96.9)	0.84
	Yes	135(3.2)	109(3.2)	26(3.1)	
Know the places to HIV testing	No	1306(28.4)	1254(34.2)	52(7.4)	p<0.001
	Yes	3761(71.6)	2723(65.8)	1038(92.6)	
Chewing khat	No	4978(84.2)	3984(84.1)	994(84.5)	
	Yes	581(15.8)	465(15.9)	116(15.5)	0.89
Alcohol drinking	No	3611(60.1)	3002(61.9)	609(53.2)	0.005
	Yes	1948(39.9)	1447(38.1)	501(46.8)	

### Uptake of premarital HIV testing among women who had autonomous versus non autonomous marriage in Ethiopia

**Fig 2:**
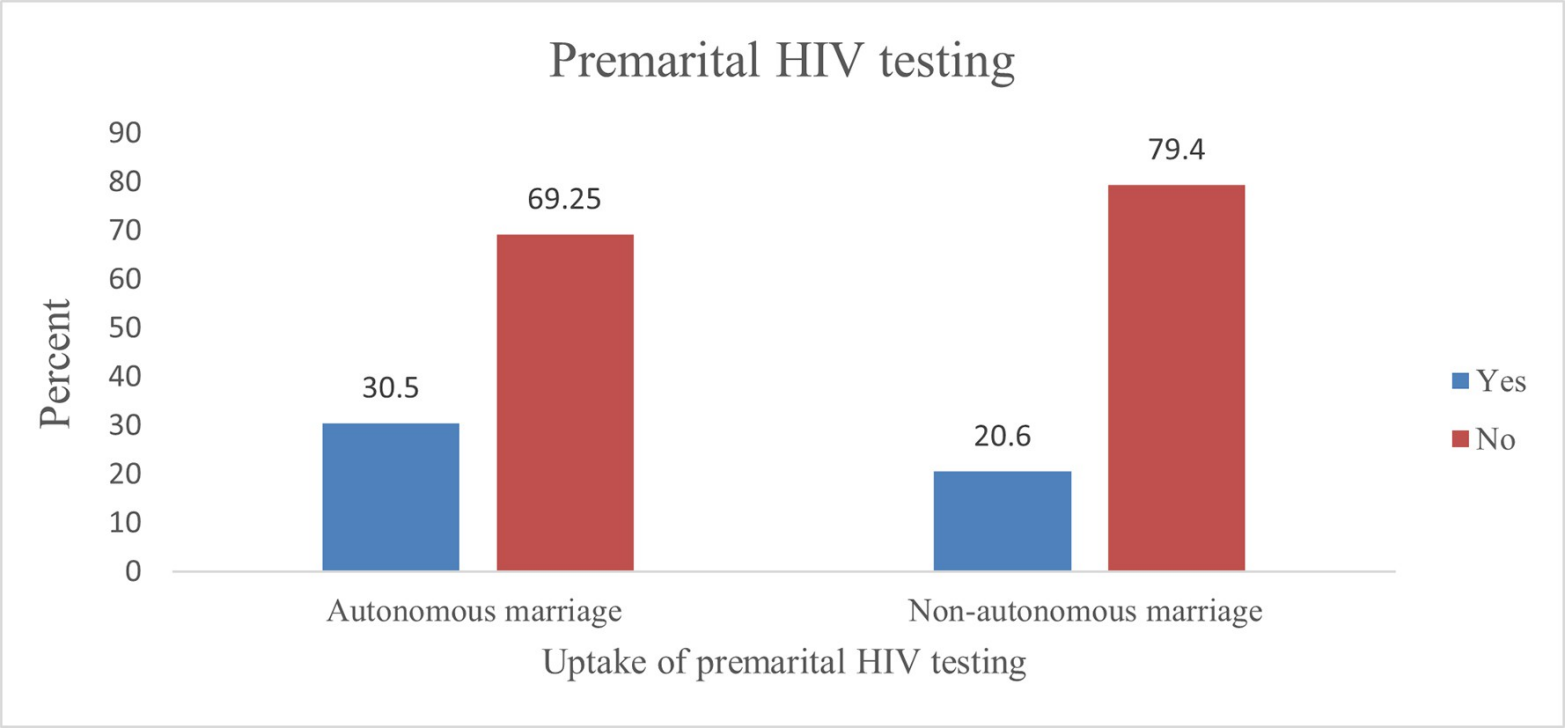
Uptake of premarital HIV testing among women who had autonomous versus non autonomous marriage in Ethiopia, EDHS 2016.

Among women who had autonomous marriage, the prevalence of premarital HIV testing was 30.5% (95% CI: 27.7–33.4). Similarly, among women who had non-autonomous marriages, the prevalence of premarital HIV testing was 20.6% (95% CI: 18.5–22.8) (Fig 2).

### Factors associated with premarital HIV testing among women who had autonomous versus non-autonomous marriage in Ethiopia

All the variables were entered into multivariable logistic regression analysis. In both groups, being an urban resident, education attainment (primary, secondary, higher), media access, being rich and richest, knowing the places for HIV testing, chewing chat, and drinking alcohol were significantly associated with premarital HIV testing. No differences in associated factors were found between women who had autonomous versus non autonomous marriage to uptake HIV testing **(Table 3).**

**Table 3:** Multivariable analysis table for identifying factors of premarital HIV testing among women who had autonomous (n = 4043) versus non autonomous (n = 5559) marriage in Ethiopia.

Variables	Category	Premarital HIV testing among women who had autonomous marriage		Premarital HIV testing among women who had not autonomous marriage	
		COR(95% CI)	AOR(95% CI)	COR(95% CI)	AOR(95% CI)
Residence	Urban	6.43(4.84–8.53)	1.94(1.28–2.93)*	4.72(3.31–6.76)	1.98(1.18–3.31)*
	Rural	1		1	1
Age	15–19	1	1	1	1
	20–24	1.73(1.12–2.68)	1.01(0.67–1.80)	0.85(0.59–1.23)	0.74(0.49–1.12)
	25–29	1.34(0.85–2.11)	0.79(0.49–1.29)	0.79(0.55–1.15)	0.72(0.46–1.10)
	30–34	1.08(0.66–1.76)	0.69(0.36–1.33)	0.45(0.30–0.66)	0.38(0.24–1.60)
	35–39	0.58(0.34–0.97)	0.37(0.18–1.73)	0.29(0.19–0.45)	0.22(0.14–1.35)
	40–44	0.41(0.22–0.75)	0.21(0.09–1.45)	0.19(0.12–0.31)	0.14(0.08–1.25)
	45–49	0.25(0.11–0.58)	0.09(0.03–1.32)	0.23(0.13–0.41)	0.19(0.09–1.39)
Educational status	No education	1	1	1	1
	Primary	4.69(3.48–6.33)	2.25(1.58–3.20)*	2.51(2.03–3.10)	1.42(1.12–1.79)*
	Secondary	12.9(8.83–19.1)	3.19(2.07–4.91)*	10.2(6.68–15.4)	2.97(1.83–4.83)*
	Higher	19.5(12.5–30.5)	4.05(2.38–6.89)*	22.8(9.71–53.7)	5.82(2.38–14.2)*
Occupation	Unemployed	1	1	1	1
	Agricultural	0.82(0.56–1.21)	1.34(0.86–2.07)	0.85(0.67–1.08)	0.85(0.65–1.11)
	Non-agricultural	2.08(1.61–2.70)	1.01(0.74–1.35)	1.67(1.27–2.17)	0.98(0.74–1.30)
Access to media	No	1	1	1	1
	Yes	5.70(4.45–7.30)	1.52(1.06–2.16)*	2.56(2.07–3.19)	1.42(1.11–1.81)*
Wealth index	Poorest	1	1	1	1
	Poorer	1.51(0.88–2.58)	1.11(0.64–1.91)	1.27(0.94–1.73)	1.04(0.73–1.47)
	Middle	1.93(1.17–3.17)	1.07(0.65–1.79)	1.67(1.18–2.36)	1.37(0.94–2.01)
	Richer	3.32(2.06–5.37)	1.53(0.90–2.61)	2.07(1.51–2.86)	1.57(1.08–2.28)*
	Richest	10.8(7.02–16.7)	1.87(1.01–3.46)*	5.25(3.67–7.51)	1.71(1.10–2.66)*
Comprehensive knowledge about HIV	No	1	1	1	1
	Yes	0.43(0.19–0.93)	0.86(0.44–1.69)	0.94(0.54–1.66)	1.64(0.93–2.92)
Know the places to HIV testing	No	1	1	1	1
	Yes	10.7(6.23–18.7)	4.87(2.69–8.79)*	6.48(4.08–10.3)	5.55(3.49–8.85)*
Chewing chat	No	1	1	1	1
	Yes	0.76(0.51–1.14)	1.21(1.12–1.88)*	1.25(1.03–1.53)*	1.75(1.08–2.85)*
Alcohol drinking	No	1	1	1	1
	Yes	2.69(2.03–3.58)	1.52(1.12–2.06)*	1.43(1.12–1.84)	1.56(1.19–2.02)*

*Statistically significant at a p-value of <0.05.

## Discussion

The study aimed to assess uptake of premarital HIV testing and associated factors among women who had autonomous versus non autonomous marriage in Ethiopia. The study finding showed that 10% more women in autonomous marriage tested for HIV relative to women in non-autonomous marriage. This may be due to the effect of being engaged in autonomous marriage for developing self-rated health. But, this finding also require further studies. Reside in an urban area, being educated, access to media, improved wealth index, knowing the place for HIV testing, chewing chat, and drinking alcohol positively predicts HIV testing in both groups. This showed that no differences in associated factors were appreciated between women who had autonomous versus non autonomous marriage to uptake HIV testing.

Considering residence, women who were residing in urban areas have higher odds to undertake premarital HIV testing. This finding is consistent with a study done in Malawi [24], and Nigeria [25, 26]. The reason for this may be better availability and accessibility of HIV testing facilities in urban settings.

The study further showed that women who were educated have higher odds to carry out premarital HIV testing. This finding is in line with a study conducted in Kenya [27], and Uganda [28]. This could be elucidated by educated women take care of HIV infection, as they easily understood both the transmission and prevention methods [28].

Regarding media access and wealth index, women who had media access and were richer and richest have higher odds to undertake premarital HIV testing. This could be expounded by the possibility that higher income for women enhances their status in the household, enables them to be educated, and can help to have better access to media easily without constraints [28].

The odds of HIV testing were higher among women who knew the place for HIV testing. This finding contrasts with a study conducted in Gambela region, which is found in Ethiopia [29]. This discrepancy may be due to sampling size variation, in which the current study was done based on nationally representative data.

As well, the present study revealed that premarital HIV testing was higher among khat chewers and alcohol drinkers compared to their counterparts. This could be due to risky sexual behavior after alcohol and khat chewing. This may have increased perceived susceptibility to HIV which in turn leads them to be tested for HIV [30, 31].

### Strength and limitation of the study

Although findings in this study are useful for policy, there are some noteworthy limitations. Since, the data were extracted from secondary data, the issue of under or over reporting of the study outcomes may be evident. Notwithstanding these limitations, this is the only study done using a nationally representative dataset to assess the uptake of premarital HIV testing and its factors among women who had autonomous versus non autonomous marriage in Ethiopia up to date.

## Conclusions

The paper shows that 10% more women in autonomous marriage tested for HIV relative to non-autonomous women. No differences in associated factors were found between women who had autonomous versus non autonomous marriage to uptake HIV testing. Being an urban resident, educated, having access to media, household wealth and knowledge of testing facilities significantly predict HIV testing among women in Ethiopia. The paper recommends that the Ethiopian government shall encourage autonomous marriage, expand access to education among women while improving their access to media to enhance their socioeconomic wellbeing and health to augment premarital HIV testing. Future researches should focus on the effect of autonomous marriage on health care service utilization on a strong study design.

## References

[1] Chen N.E., Meyer J.P., and Springer S.A., Advances in the prevention of heterosexual transmission of HIV/AIDS among women in the United States. Infectious disease reports, 2011. 3(1). doi: 10.4081/idr.2011.e6 23745166 PMC3671603

[2] Nicolosi A., et al., The efficiency of male-to-female and female-to-male sexual transmission of the human immunodeficiency virus: a study of 730 stable couples. Epidemiology, 1994: p. 570–575. doi: 10.1097/00001648-199411000-00003 7841237

[3] Campbell C.A., Women, families and HIV/AIDS: A sociological perspective on the epidemic in America. 1999: Cambridge University Press.

[4] Kates J., et al., A Report on Women and HIV/AIDS in the US. The Kaiser Family Foundation. http://kaiserfamilyfoundation.files.wordpress.com/2013/04/8436.pdf. Retrieved, 2013. 5: p. 12–13.

[5] Magadi M.A., Understanding the gender disparity in HIV infection across countries in sub-Saharan Africa: evidence from the Demographic and Health Surveys. Sociol Health Illn, 2011. 33(4): p. 522–39. doi: 10.1111/j.1467-9566.2010.01304.x 21545443 PMC3412216

[6] Central Statistical Agency (CSA) [Ethiopia] and ICF. Ethiopia Addis Ababa, E., and Rockville, Maryland, USA, CSA, and ICF, Ethiopia Demographic and Health Survey. 2018.

[7] Altman R., et al., Premarital HIV-1 testing in New Jersey. Journal of acquired immune deficiency syndromes, 1992. 5(1): p. 7–11. 1738089

[8] DeZoysa I., et al., The Voluntary HIV-1 Counseling and Testing Efficacy Study Group. Efficacy of voluntary HIV-1 counselling and testing in individuals and couples in Kenya, Tanzania, and Trinidad: a randomised trial. Lancet, 2000. 356: p. 103–12. 10963246

[9] Ganczak M., The impact of premarital HIV testing: a perspective from selected countries from the Arabian Peninsula. AIDS care, 2010. 22(11): p. 1428–1433. doi: 10.1080/09540121003692615 20936541

[10] MOH, Guidelines for HIV counselling and testing in Ethiopia. 2007, Ministry of Health Addis Ababa.

[11] McKillip J., The effect of mandatory premarital HIV testing on marriage: the case of Illinois. American Journal of Public Health, 1991. 81(5): p. 650–653. doi: 10.2105/ajph.81.5.650 2014872 PMC1405095

[12] Cleary P.D., et al., Compulsory premarital screening for the human immunodeficiency virus: Technical and public health considerations. JAMA, 1987. 258(13): p. 1757–1762. 3476759

[13] Barmania S. and Aljunid S.M., Premarital HIV testing in Malaysia: a qualitative exploratory study on the views of major stakeholders involved in HIV prevention. BMC international health and human rights, 2017. 17(1): p. 1–10. 28490382 10.1186/s12914-017-0120-8 PMC5424423

[14] Dunkle K.L., et al., New heterosexually transmitted HIV infections in married or cohabiting couples in urban Zambia and Rwanda: an analysis of survey and clinical data. The Lancet, 2008. 371(9631): p. 2183–2191. doi: 10.1016/S0140-6736(08)60953-8 18586173

[15] Campbell M.S., et al., Viral linkage in HIV-1 seroconverters and their partners in an HIV-1 prevention clinical trial. PloS one, 2011. 6(3): p. e16986. doi: 10.1371/journal.pone.0016986 21399681 PMC3047537

[16] Matovu J.K., Preventing HIV transmission in married and cohabiting HIV-discordant couples in sub-Saharan Africa through combination prevention. Current HIV research, 2010. 8(6): p. 430–440. doi: 10.2174/157016210793499303 20636280

[17] Rennie S. and Mupenda B., Ethics of mandatory premarital HIV testing in Africa: the case of Goma, Democratic Republic of Congo. Developing world bioethics, 2008. 8(2): p. 126–137. doi: 10.1111/j.1471-8847.2007.00199.x 19143089 PMC4681495

[18] Organization W.H., Statement on HIV testing and counseling: WHO, UNAIDS re-affirm opposition to mandatory HIV testing. Found at: http://www.who.int/hiv/events/2012/world_aids_day/hiv_testing_counselling/en, 2012.

[19] ICF, C.S.A.a., Ethiopia Demographic and Health Survey 2016. 2016.

[20] Ahmed M. and Seid A., Factors associated with premarital HIV testing among married women in Ethiopia. PloS one, 2020. 15(8): p. e0235830. doi: 10.1371/journal.pone.0235830 32745083 PMC7398550

[21] Global A., New insights on preventing child marriage. 2007.

[22] Koenig M.A., et al., Coerced first intercourse and reproductive health among adolescent women in Rakai, Uganda. International family planning perspectives, 2004: p. 156–163. doi: 10.1363/3015604 15590381

[23] Khawaja M. and Hammoury N., Coerced sexual intercourse within marriage: a clinic-based study of pregnant Palestinian refugees in Lebanon. Journal of midwifery & women’s health, 2008. 53(2): p. 150–154. doi: 10.1016/j.jmwh.2007.09.001 18308266

[24] Misiri H. and Muula A.S., Attitudes towards premarital testing on human immunodeficiency virus infection among Malawians. Croat Med J, 2004. 45(1): p. 84–7. 14968460

[25] Ibrahim M., et al., Socio-demographic determinants of HIV counseling and testing uptake among young people in Nigeria. International Journal of Prevention and Treatment, 2013. 2(3): p. 23–31.

[26] Oginni A., Obianwu O., and Adebajo S., Socio-demographic Factors Associated with Uptake of HIV Counseling and Testing (HCT) among Nigerian Youth. AIDS research and human retroviruses, 2014. 30(S1): p. A113–A113.

[27] Onsomu E.O., et al., Importance of the media in scaling-up HIV testing in Kenya. SAGE Open, 2013. 3(3): p. 2158244013497721.

[28] Fabiani M., et al., Investigating factors associated with uptake of HIV voluntary counselling and testing among pregnant women living in North Uganda. AIDS care, 2007. 19(6): p. 733–739. doi: 10.1080/09540120601087731 17573592

[29] Kebede D., et al., Khat and alcohol use and risky sex behaviour among in-school and out-of-school youth in Ethiopia. BMC public health, 2005. 5(1): p. 109. doi: 10.1186/1471-2458-5-109 16225665 PMC1274331

[30] Abebe D., et al., Khat chewing habit as a possible risk behaviour for HIV infection: A case-control study. Ethiopian Journal of Health Development, 2005. 19(3): p. 174–181.

[31] Seme A., Mariam D.H., and Worku A., The association between substance abuse and HIV infection among people visiting HIV counselling and testing centres in Addis Ababa, Ethiopia. Ethiopian Journal of Health Development, 2005. 19(2): p. 116–125.

