# Supplementary material for: Uptake of premarital HIV testing and associated factors among women who had autonomous versus non autonomous marriage in Ethiopia: A nationwide study
Source: PLoS One. 2022 Aug 18;17(8):e0271879. doi: 10.1371/journal.pone.0271879 (PMC9387857; doi:10.1371/journal.pone.0271879)
Supplement: Attachment — Submitted filename: PMHT Ethiopia.docx [file pone.0271879.s001.docx]

**Does the magnitude of premarital HIV testing differ among women who had autonomous versus nonautonomous marriage in Ethiopia? Evidence from a nationwide household survey**

**Reviewer’s report**

I’d like to thank the editors for giving me the opportunity to review the manuscript and I congratulate the authors for their work. The authors used data from a nationally representative survey to determine the extent to which women were tested for HIV prior to marriage in Ethiopia. I doubt whether the paper can be published in its current form. Some sections of the manuscript may not be clear to potential readers. I’d suggest that the authors provide their revised manuscript to someone with strong English Language proficiency to read the manuscript and correct some typing and grammatical errors prior to resubmission. The following comments and suggestions could help improve the quality of manuscript:

INTRODUCTION

Paragraph 3: The authors cited a study conducted in Ethiopia. What was the target group for the cited study? Was it for married women? Please specify.

Paragraph 4: The authors should define autonomous and non-autonomous marriage to their readers. If the definition is provided at this stage, they don’t need to repeat the definition in the subsequent sections of the manuscript.

Paragraph 5: Based on my review of this manuscript and comments in the findings/results section (see below), I suggest that the authors reformulate the objectives of their analysis. The following could serve as a guide: The objectives of this analysis were: 1) To estimate the prevalence of pre-marital HIV testing (PMHT) among married women in Ethiopia; 2) To determine whether there is any significant difference in the prevalence of PMHT among women who had autonomous vs non-autonomous marriage, 3) To investigate the factors associated with PMHT among married women in Ethiopia.

Since data were derived from a nationally representative population-based survey, I suggest that authors use prevalence rather than magnitude. In the last sentence of the introduction section, the authors should briefly outline the relevance and usefulness of their study findings.

**METHODS and material section**

While we understand that the detailed methods are found in the EHDS report, the authors didn’t outline how cluster sampling technique was conducted. They simply stated that: “The first and second stages involved the selection of clusters and households in each cluster, respectively” This is too brief. The authors should outline (briefly) how the first stage and second stage sampling was conducted then reference the EDHS report for more details. In the last sentence of paragraph 1 (Methods section), the authors should indicate the number of households that participated in the EDHS.

Selection criteria: If the authors define the different types of marriage in the Introduction section, they don’t need to repeat it here. I suggest that the definition in brackets should be deleted.

**Study Variables**

The authors did not explain how their dependent variable (pre-marital HIV testing) was assessed in the survey. Please kindly include this in your manuscript so that readers can understand how the variable was evaluated. Furthermore, the explanation of how marriage was classified as autonomous and non-autonomous is not clearly elaborated. The authors should rewrite the sentence.

**Independent variables**: The authors should indicate how the variables: alcohol drinking and wealth index were assessed in the survey.

It is not clear how the authors determined women with or without comprehensive knowledge of HIV status. Please kindly explain how this was done.

**Statistical Analysis**

Was the data set used in this analysis already weighted by the EDHS team before being provided to the authors or it was unweighted data? If unweighted, the authors should provide details of the sampling design analysis so that readers should understand how this was done. The authors simply stated that “All statistical procedures incorporated complex sampling design analysis applied in the 2016 EDHS.” In my view, this statement doesn’t explain to the readers how this was done.

The authors should conduct Chi-square test to determine whether there were differences in demographic characteristics among the 2 groups of married women.

The authors indicated that …” *All independent variables were entered in the multivariable logistic regression model irrespective of the p-values in the bivariate analysis.”* Could you explain to your readers the essence of conducting a bivariate association despite the fact that you still included all independent variables in the model. Did the authors test for multicollinearity prior to multivariate analysis? If not, why? What was your level of statistical significance? Where the tests conducted 2-sided? Please provide details to your readers.

**FINDINGS**

The findings of this analysis shows that the factors associated with pre-marital HIV testing (PMHT) among women with autonomous marriage were the same for women with non-autonomous marriage. Consequently, I think there was no added value examining and presenting these same factors in different tables for both groups of women. This could be considered as repeating the analysis without any new information and perhaps explains why there are lots of data/tables in the manuscript.

If the analysis had showed that the factors were substantially different among the 2 sub-groups, then it would have been interesting to conduct a sub-group analysis and report the results.

As a result, I suggest that the authors reanalyze the pooled data (combining both groups) and investigate the factors associated with PMHT among married women in Ethiopia. The authors main comparison should focus on investigating whether there was a significant difference in the prevalence of PMHT among women who had autonomous vs non-autonomous marriages. If there is a difference, the authors should provide an explanation (in the Discussion Section) for the observed results taking into consideration the socio-cultural context of Ethiopia.

**Discussion**

The authors cited studies conducted in Kenya, Uganda, Malawi, and Nigeria to compare and contrast the findings of their study. What was the target group for these studies? Was the target group married women or women with autonomous/non-autonomous marriages. It is appropriate that the authors cite studies conducted among the same target population while comparing and contrasting their findings. Please consider this as you revise your manuscript.

**Limitations**

The authors outline only one limitation of their analysis. Furthermore, the statement on “difficult to find causal relationships due to contemporaneous ……”is not clear to their readers. Please provide details so that it should be clear to your readers.

What about limitations associated with the fact that responses for PMHT were self-reported? Could the participants have under-reported or over-reported that they received HIV testing before marriage? Was there any limitation associated with secondary data used in the analysis? Please provide details of the limitations of the analysis. What do the authors recommend for future studies in other settings?

In what ways do you think the findings of this analysis/study could help inform HIV testing policies and guidelines in Ethiopia and beyond especially with regards to PMHT.
