# Supplementary material for: Uptake of premarital HIV testing and associated factors among women who had autonomous versus non autonomous marriage in Ethiopia: A nationwide study
Source: PLoS One. 2022 Aug 18;17(8):e0271879. doi: 10.1371/journal.pone.0271879 (PMC9387857; doi:10.1371/journal.pone.0271879)
Supplement: Attachment — Submitted filename: Review comments - 2.docx [file pone.0271879.s002.docx]

Review comments:

**General remarks:**

I thank the authors for their efforts to address the comments. The manuscript is now in better shape than the first version.

**Title**

The title is clear and informative.

I suggest the authors remove the word, ‘its’ just before associated factors

**Abstract**

***Background:***

The background does not underscore the study gap. This should come out clearly to inform your study aim.

***Method:***

Good

***Results:***

The result section should report on the demographics first (how many women were in the category of autonomous versus nonautomonous marriage) before you report on the other objectives. Include predictors as part of the results section.

The last sentence is missing a word (of)

Do you mean residence in a rural area?

***Conclusion:***

The opening words are redundant….The study concludes that

Numbers ten and above should be written in Arabic numeral e.g. 10% and not in words (ten percent)

The paper recommends that the……is also redundant

Please use active voice while writing your abstract

**Introduction**

The introduction section is clear and much focused except for some grammatical issues.

I suggest the authors enlist the support of a native English to proofread the paper or English editing software.

I prefer you state the purpose of the study instead of the objectives in the last paragraph.

**Methods and Materials**

Give a reference for your data analysis software, SPSS.

The operational definitions of knowledge about HIV and media access could be entered as new paragraphs without bolding the words

**Results**

The second, third, and fourth sentences of the Results section have major grammatical issues that need to be addressed.

We use the terminology study participants NOT study subject.

Avoid the phrase, ‘same fashion’. Scholarly writing demands scholar words and or phrases

I like figure but it needs to be well formatted with the same word style and font as the entire document. Also, remove the longitudinal lines.

Since the p-values are not included in Table 3, you could quote the p-values in your description of the significant factors in the text.

**Discussion**

I am not comfortable with the opening paragraph of your discussion. You could start this section by saying…..We aimed to assess the prevalence of HIV testing and associated factors among……. We found that…..

Then, you go ahead and pick each component of this summary and discuss following the earlier guidelines

For each of the discussion paragraphs, you should: a) State your findings (interpret the results), b) explain your findings c) compare and contrast findings with previous literature d) Justify differences in findings if any e) state the public health significance of your findings

The second sentence under strengths and limitations is not clear. Please revise this.

**Conclusion**

Have a sentence that makes suggestions for the area(s) of future research

Also, make a recommendation for autonomous marriage.
