# Supplementary material for: Uptake of premarital HIV testing and associated factors among women who had autonomous versus non autonomous marriage in Ethiopia: A nationwide study
Source: PLoS One. 2022 Aug 18;17(8):e0271879. doi: 10.1371/journal.pone.0271879 (PMC9387857; doi:10.1371/journal.pone.0271879)
Supplement: Attachment — Submitted filename: Reviewers Report.docx [file pone.0271879.s003.docx]

**Reviewer’s report**

**Uptake of premarital HIV testing and its associated factors among women who had autonomous versus non autonomous marriage in Ethiopia: a nationwide study.**

I have reviewed for the second time the manuscript titled: “Uptake of premarital HIV testing and its associated factors among women who had autonomous versus non autonomous marriage in Ethiopia: a nationwide study.” While the authors have responded to the initial comments, it is my view that this paper can not be published in its current form. There are several typos and grammatical errors which need to be corrected. Sharing the manuscript to an English native speaker for review and correction of these errors will be very helpful. Furthermore, there are other methodological aspects that could be addressed to improve the quality of the manuscript.

Comments:

Page 1

Line 4. For the sake of coherence, I suggest that the 3^rd^ sentence in the first paragraph (“In Ethiopia….” ) should appear immediately after the 1^st^ sentence.

Page 1

Paragraph 3: Correct the typo by using capitalization. It should be written as Ethiopia Demographic Health Survey”, not Ethiopia demographic health Survey (EDHA). There are many similar typos and grammatical errors in the manuscript that should be corrected.

Methods section:

In the selection criteria, the authors should insert a comma for numbers which are more than 999. Hence, “9602” should be written as “9,602”. Please carefully review the manuscript and correct all similar typos including those in Figure 1.

I think it would be helpful to the readers if the authors insert the % of women for each subgroup. In the sentence, “ From the included sample, 4043 of the women had autonomous marriage, and 5559 of the women had non-autonomous marriage” could be written as “From the included sample, 4,043 (42.1%) of the women had autonomous marriage, and 5,559(57.9%) of the women had non-autonomous marriage”.

Study variables:

The authors should include the question that was asked to evaluate whether women undertook premarital HIV testing before marriage. A statement such as: [Respondents were asked “Did you undertake an HIV test before you got married?” The responses options were “Yes” or “No”]. This is an example, the authors should correct the manuscript using the exact question that was asked in the survey.

Access to media: The authors should indicate the response options that were used in the survey. For example, respondents were asked: “How often do you, read a newspaper, listen to the radio, or watch TV in a week?” What were the other response options aside from “once a week”? The authors should provide adequate information, so that researchers in other countries can replicate the study.

Results

I suggest that instead of the title: “Descriptive statistics of women who had autonomous versus non-autonomous marriage”, the authors should say “Socio-demographic characteristics of study participants by type of marriage”

Under the subheading: “Uptake of premarital HIV testing among women who had autonomous versus non autonomous marriage in Ethiopia”, the authors write: “Among 4043 women who had autonomous marriage, the magnitude of premarital HIV testing”. I suggest that the authors replace the word “magnitude” with “prevalence” as previously suggested.

This analysis shows that there wasn’t any relevance of conducting a subgroup analysis of the factors associated with premarital HIV among autonomous vs non-autonomous marriage because the factors identified are the same for both groups. What additional value does this add? If the government of a country has enacted a policy that women should undertake HIV testing before marriage, the decision to take the test is not influenced by the type of marriage (autonomous vs non-autonomous marriage.) Therefore, whether the marriage is autonomous vs non-autonomous, women are expected to undergo HIV testing.

It is this reviewer’s view that the central focus of this article which unfortunately the authors did not take advantage was to estimate the % of women who underwent HIV testing before marriage, determine whether there was any statistically significant difference in the % of women who received HIV testing before marriage among the 2 groups and then examine the factors associated with premarital HIV testing among married women in Ethiopia in a pooled multivariate logistic model consisting of both samples. I will therefore suggest that the authors conduct a pooled analysis.

In the discussion section, the authors compared and contrasted their findings with studies that examined premarital HIV status among married women. There was very little or no discussion on autonomous vs non-autonomous marriage, which demonstrates that that there wasn’t any new or interesting finding from the subgroup analysis (autonomous and non-autonomous marriage.)

The argument the authors are making is that the factors affecting premarital HIV testing among married women in Ethiopia is known, but that their study was focused on estimating the prevalence of premarital HIV testing and its associated factors independently among the two groups to appreciate the differences. Unfortunately, the factors were the same, which suggest that there no value of separating them.

If the subgroup analysis generated different results/factors associated with…., that would have justified their approach.
