# Supplementary material for: Uptake of premarital HIV testing and associated factors among women who had autonomous versus non autonomous marriage in Ethiopia: A nationwide study
Source: PLoS One. 2022 Aug 18;17(8):e0271879. doi: 10.1371/journal.pone.0271879 (PMC9387857; doi:10.1371/journal.pone.0271879)
Supplement: Attachment — Submitted filename: Point by point response for reviewers.docx [file pone.0271879.s004.docx]

Thank you very much for PLOS one editorial office, academic editors, as well as reviewers of this manuscript entitled ***uptake of premarital HIV testing and associated factors among women who had autonomous versus non autonomous marriage in Ethiopia: a nationwide study.***

The written documents below explained point by point response for respective reviewer’s comment.

**Reviewer 1 comments and authors response:**

Reviewer #1: I thank the authors for addressing my comments.
However, they should do a final grammar check; for instance, the first sentence of the abstract has a mixing of past and present tenses. The authors should change the word "faced" to "face".
Similarly, the ethics approval statement should be stated in the past tense, not the present tense.

**Author response: All the comments are addressed in the submitted revised manuscript.**

**Reviewer 2 comments and authors response:**

Reviewer #2: I have reviewed for the second time the manuscript titled: “Uptake of premarital HIV testing and its associated factors among women who had autonomous versus non autonomous marriage in Ethiopia: a nationwide study.” While the authors have responded to the initial comments, it is my view that this paper can not be published in its current form. There are several typos and grammatical errors which need to be corrected. Sharing the manuscript to an English native speaker for review and correction of these errors will be very helpful. Furthermore, there are other methodological aspects that could be addressed to improve the quality of the manuscript.
Comments:
Page 1
Line 4. For the sake of coherence, I suggest that the 3rd sentence in the first paragraph (“In Ethiopia….” ) Should appear immediately after the 1st sentence.

**Author response: The authors amended the manuscript based on the reviewers comment.**
Page 1
Paragraph 3: Correct the typo by using capitalization. It should be written as Ethiopia Demographic Health Survey”, not Ethiopia demographic health Survey (EDHA). There are many similar typos and grammatical errors in the manuscript that should be corrected.
**Author response: The authors amended the manuscript based on the reviewers comment.**

Methods section:
In the selection criteria, the authors should insert a comma for numbers which are more than 999. Hence, “9602” should be written as “9,602”. Please carefully review the manuscript and correct all similar typos including those in Figure 1.
**Author response: The authors amended the manuscript based on the reviewers comment.**

I think it would be helpful to the readers if the authors insert the % of women for each subgroup. In the sentence, “ From the included sample, 4043 of the women had autonomous marriage, and 5559 of the women had non-autonomous marriage” could be written as “From the included sample, 4,043 (42.1%) of the women had autonomous marriage, and 5,559(57.9%) of the women had non-autonomous marriage”.

**Author response: The authors amended the manuscript based on the reviewers comment.**
Study variables:
The authors should include the question that was asked to evaluate whether women undertook premarital HIV testing before marriage. A statement such as: [Respondents were asked “Did you undertake an HIV test before you got married?” The responses options were “Yes” or “No”]. This is an example, the authors should correct the manuscript using the exact question that was asked in the survey.

**Author response: The authors amended the manuscript based on the reviewers comment.**

Access to media: The authors should indicate the response options that were used in the survey. For example, respondents were asked: “How often do you, read a newspaper, listen to the radio, or watch TV in a week?” What were the other response options aside from “once a week”? The authors should provide adequate information, so that researchers in other countries can replicate the study.

**Author response: The authors amended the manuscript based on the reviewers comment.**

Results
I suggest that instead of the title: “Descriptive statistics of women who had autonomous versus non-autonomous marriage”, the authors should say “Socio-demographic characteristics of study participants by type of marriage”

**Author response: The authors amended the manuscript based on the reviewers comment.**

Under the subheading: “Uptake of premarital HIV testing among women who had autonomous versus non autonomous marriage in Ethiopia”, the authors write: “Among 4043 women who had autonomous marriage, the magnitude of premarital HIV testing”. I suggest that the authors replace the word “magnitude” with “prevalence” as previously suggested.

**Author response: The authors amended the manuscript based on the reviewers comment.**

This analysis shows that there wasn’t any relevance of conducting a subgroup analysis of the factors associated with premarital HIV among autonomous vs non-autonomous marriage because the factors identified are the same for both groups. What additional value does this add? If the government of a country has enacted a policy that women should undertake HIV testing before marriage, the decision to take the test is not influenced by the type of marriage (autonomous vs non-autonomous marriage.) Therefore, whether the marriage is autonomous vs non-autonomous, women are expected to undergo HIV testing.

It is this reviewer’s view that the central focus of this article which unfortunately the authors did not take advantage was to estimate the % of women who underwent HIV testing before marriage, determine whether there was any statistically significant difference in the % of women who received HIV testing before marriage among the 2 groups and then examine the factors associated with premarital HIV testing among married women in Ethiopia in a pooled multivariate logistic model consisting of both samples. I will therefore suggest that the authors conduct a pooled analysis.

**Author response: The intention of this paper is to see whether the factors are different or not between the two groups. The pooled analysis is already done in the previous published research paper. Even if the factors among the sub groups are the same, the strength of the association is different.**

In the discussion section, the authors compared and contrasted their findings with studies that examined premarital HIV status among married women. There was very little or no discussion on autonomous vs non-autonomous marriage, which demonstrates that that there wasn’t any new or interesting finding from the subgroup analysis (autonomous and non-autonomous marriage.)

**Author response: The authors amended the manuscript based on the reviewers comment.**

**Reviewer 3 comments and authors response:**

Reviewer #3: General remarks:
I thank the authors for their efforts to address the comments. The manuscript is now in better shape than the first version.
Title
The title is clear and informative.
I suggest the authors remove the word, ‘its’ just before associated factors

**Author response: The authors amended the manuscript based on the reviewers comment.**
Abstract
Background:
The background does not underscore the study gap. This should come out clearly to inform your study aim.

**Author response: The authors amended the manuscript based on the reviewers comment.**
Method:
Good
Results:
The result section should report on the demographics first (how many women were in the category of autonomous versus nonautomonous marriage) before you report on the other objectives. Include predictors as part of the results section.
The last sentence is missing a word (of)
Do you mean residence in a rural area?

**Author response: The authors amended the manuscript based on the reviewers comment.**
Conclusion:
The opening words are redundant….The study concludes that
Numbers ten and above should be written in Arabic numeral e.g. 10% and not in words (ten percent)
The paper recommends that the……is also redundant
Please use active voice while writing your abstract

**Author response: The authors amended the manuscript based on the reviewers comment.**

Introduction
The introduction section is clear and much focused except for some grammatical issues.
I suggest the authors enlist the support of a native English to proofread the paper or English editing software.
I prefer you state the purpose of the study instead of the objectives in the last paragraph.

**Author response: The authors amended the manuscript based on the reviewers comment.**

Methods and Materials
Give a reference for your data analysis software, SPSS.
The operational definitions of knowledge about HIV and media access could be entered as new paragraphs without bolding the words

**Author response: The authors amended the manuscript based on the reviewers comment.**

Results
The second, third, and fourth sentences of the Results section have major grammatical issues that need to be addressed.
We use the terminology study participants NOT study subject.
Avoid the phrase, ‘same fashion’. Scholarly writing demands scholar words and or phrases
I like figure but it needs to be well formatted with the same word style and font as the entire document. Also, remove the longitudinal lines.
Since the p-values are not included in Table 3, you could quote the p-values in your description of the significant factors in the text.

**Author response: The authors amended the manuscript based on the reviewers comment.**
Discussion
I am not comfortable with the opening paragraph of your discussion. You could start this section by saying…..We aimed to assess the prevalence of HIV testing and associated factors among……. We found that…..
Then, you go ahead and pick each component of this summary and discuss following the earlier guidelines
For each of the discussion paragraphs, you should: a) State your findings (interpret the results), b) explain your findings c) compare and contrast findings with previous literature d) Justify differences in findings if any e) state the public health significance of your findings
The second sentence under strengths and limitations is not clear. Please revise this.

**Author response: The authors amended the manuscript based on the reviewers comment.**
Conclusion
Have a sentence that makes suggestions for the area(s) of future research
Also, make a recommendation for autonomous marriage.

**Author response: The authors amended the manuscript based on the reviewers comment.**

**Thank you for reviewer’s constructive comments**
