# Supplementary material for: Uptake of premarital HIV testing and associated factors among women who had autonomous versus non autonomous marriage in Ethiopia: A nationwide study
Source: PLoS One. 2022 Aug 18;17(8):e0271879. doi: 10.1371/journal.pone.0271879 (PMC9387857; doi:10.1371/journal.pone.0271879)
Supplement: Attachment — Submitted filename: Response to reviewers.docx [file pone.0271879.s005.docx]

Thank you very much for PLOS one editorial office, academic editors, as well as reviewers of this manuscript entitled **uptake of premarital HIV testing and associated factors among women who had autonomous versus non autonomous marriage in Ethiopia: a nationwide study.**

The written documents below explained point by point response for respective reviewer and editor comments.

**Reviewer 1 comments and authors response:**

Please define whether you mean factors associated with premarital HIV testing among women who had autonomous versus non-autonomous marriage in Ethiopia.

**Author response**: I have included in the revised manuscript

**Editor comments and author response**

Please review your reference list to ensure that it is complete and correct. If you have cited papers that have been retracted, please include the rationale for doing so in the manuscript text, or remove these references and replace them with relevant current references. Any changes to the reference list should be mentioned in the rebuttal letter that accompanies your revised manuscript. If you need to cite a retracted article, indicate the article’s retracted status in the References list and also include a citation and full reference for the retraction notice.

**Author response**: I have reviewed the manuscript and I did not found retracted articles

**Thank you for reviewer’s and editor constructive comments**
